# Aging-Related Changes in Bimanual Coordination as a Screening Tool for Healthy Aging

**DOI:** 10.3390/geriatrics10020045

**Published:** 2025-03-17

**Authors:** Yusuke Shizuka, Shin Murata, Akio Goda, Shun Sawai, Shoya Fujikawa, Ryosuke Yamamoto, Takayuki Maru, Kotaro Nakagawa, Hideki Nakano

**Affiliations:** 1 Graduate School of Health Sciences, Kyoto Tachibana University, 34 Yamada-cho, Oyake, Yamashina-ku, Kyoto-shi 607-8175, Kyoto, Japan; h901124020@st.tachibana-u.ac.jp (Y.S.); murata-s@tachibana-u.ac.jp (S.M.); h901523005@st.tachibana-u.ac.jp (S.S.); h901123010@st.tachibana-u.ac.jp (S.F.); h901123013@st.tachibana-u.ac.jp (R.Y.); 2 Department of Rehabilitation, Kyoto Kuno Hospital, 22-500 Honmachi, Higashiyama-ku, Kyoto-shi 605-0981, Kyoto, Japan; 3 Department of Physical Therapy, Faculty of Health Sciences, Kyoto Tachibana University, 34 Yamada-cho, Oyake, Yamashina-ku, Kyoto-shi 607-8175, Kyoto, Japan; maru@tachibana-u.ac.jp (T.M.); nakagawa-k@tachibana-u.ac.jp (K.N.); 4 Department of Physical Therapy, Faculty of Health and Medical Sciences, Hokuriku University, 1-1 Taiyogaoka, Kanazawa-shi 920-1154, Ishikawa, Japan; a-goda@hokuriku-u.ac.jp; 5 Department of Rehabilitation, Tesseikai Neurosurgical Hospital, 28-1 Nakanohonmachi, Shijonawate-shi 575-8511, Osaka, Japan; 6 Department of Rehabilitation, Junshinkai Kobe Hospital, 868-37 Kozukadai, Tarumi-ku, Kobe-shi 655-0008, Hyogo, Japan; 7 Nagashima Neurosurgery Rehabilitation Clinic, 1st and 2nd Floor Niitaka Clinic Center Building, 2-3-2 Niitaka, Yodogawa-ku, Osaka-shi 532-0033, Osaka, Japan

**Keywords:** bimanual coordination, finger tapping, age-related changes, young adults, young-old adults, old-old adults

## Abstract

**Background/Objectives:** The steady increase in the global older adult population highlights critical challenges, including the development of preventive strategies to extend healthy life expectancy and support independence in activities of daily living. Although there is an aging-related reduction in manual dexterity, the difference in bimanual coordination performance between young and older adults remains unclear. We aimed to elucidate the characteristics of bimanual coordination among young, young-old, and old-old adult participants. **Methods:** The participants performed in-phase (tapping the thumb and index finger together as fast as possible) and anti-phase (alternating movement between the left and right fingers) bimanual coordination tasks, and intergroup comparison of the task parameters was performed. The receiver operating characteristic curve was also conducted to calculate age cut-off points for bimanual coordination. **Results:** The number and frequency of taps significantly decreased sequentially in young, young-old, and old-old adults, whereas the average of tap interval significantly increased in this order (*p* < 0.05). There was no significant difference between the young-old and old-old groups in the average local maximum distance (*p* > 0.05). These findings indicate that bimanual coordination task performance varies depending on specific parameters. Furthermore, the age cut-off points for bimanual coordination were determined as 68.5 years for the right-hand number of taps (AUC = 0.73) in the anti-phase task, 73.5 years for the right-hand average of tapping interval (AUC = 0.72) in the anti-phase task, and 65.5 years for the left-hand frequency of taps (AUC = 0.72) of the anti-phase task. **Conclusions:** the number of taps, average of tapping interval, and frequency of taps are potential indicators of aging-related changes in bimanual coordination.

## 1. Introduction

In recent years, the proportion of older adults in the global population has steadily increased. The United Nations World Social Report 2023 indicates that the number of people aged 65 years and above will more than double from 761 million in 2021 to 1.6 billion by 2050. Likewise, the population aged 80 years and above is expected to grow rapidly. In 2021, one in ten people in the global population was 65 years or older, and by 2050, this could increase to one in six people [1]. Compared with younger adults, older adults tend to experience a decline in physical function. Aging-related deterioration in motor function significantly contributes to increased caregiver burden, higher hospitalization rates, and increasing healthcare costs [2,3]. Consequently, interest in promoting healthy aging has been growing. Healthy aging is defined as the process of developing and maintaining functional abilities that enable wellbeing in older age [4]. Furthermore, the relationship between healthy aging and physical activity has been underscored with regard to potential contributions to pain alleviation and prevention of falls, osteoporosis, sarcopenia, and cognitive impairments [5]. Thus, healthy aging is essential for maintaining the ability to perform activities of daily living (ADL). Therefore, in the global population where the number of older adults is increasing annually, it is crucial to implement preventive measures to promote healthy aging, extend healthy life expectancy, and support the ADL independence of older adults.

The upper limbs are frequently used in daily life, and older adults perform more bimanual than unilateral tasks [6]. Therefore, both maintaining and improving finger function are crucial for older adults to lead healthy lives. Incel et al. found that grip and pinch strengths in older adults are associated with activity limitations and quality of life [7]. Moreover, maximal grip strength and motor performance involving coordinated bimanual control decrease with age, which suggests that older adults may find it challenging to perform daily activities that require bimanual movements, such as holding a bottle while simultaneously unscrewing its cap [8]. Therefore, finger function plays a vital role in ADL performance, with a particularly strong correlation between bimanual tasks and daily activity levels in older adults. Thus, to promote healthy aging and support independence in older adults, it is essential to appropriately assess finger function during bimanual movements and undertake preventive interventions to prevent aging-related decline.

A meta-analysis investigating aging-related changes in bimanual movement among older adults revealed a decline in accuracy, increased variability, and prolonged execution times for bimanual coordination [9]. Another meta-analysis investigating the characteristics of bimanual coordination in older adults found that motor performance deterioration was more pronounced in asymmetrical rather than symmetrical bimanual tasks [10]. Thus, it has been clarified that bimanual coordination performance in older adults declines in comparison to that in young adults. Furthermore, both young-old adults and old-old adults exhibit reduced bimanual performance compared to younger adults [11,12]. Asymmetrical bimanual coordination performance is lower in young adults than in older adults, with performance decline particularly pronounced in tasks that require higher movement speeds [11]. The accuracy of both symmetrical and asymmetrical bimanual force control is lower in old-old adults, compared to young adults [12]. Therefore, bimanual coordination performance, which plays a crucial role in daily activities, decreases in both young-old and old-old adults compared to that in young adults. Typically, old-old adults experience more pronounced decline in physical and cognitive functions than young adults [13,14,15]. Therefore, although aging-related changes in bimanual coordination may occur in young-old and old-old adults, the differences in bimanual coordination performance between young-old and old-old adults remain unclear.

We aimed to clarify the age-specific characteristics of bimanual coordination in young, young-old, and old-old adults. The research hypothesis was that bimanual coordination performance would be lower in older adults than in young adults and that among older adults, the decline in performance would be more pronounced in old-old adults than in young-old adults. If the age-specific characteristics of bimanual coordination can be clarified, these tasks can be applied as screening tools for health management and promotion among older adults. Moreover, early detection and intervention for the decline in ADL among older adults could contribute to promoting healthy aging and extending their health span.

## 2. Materials and Methods

### 2.1. Materials

**Figure 1:**
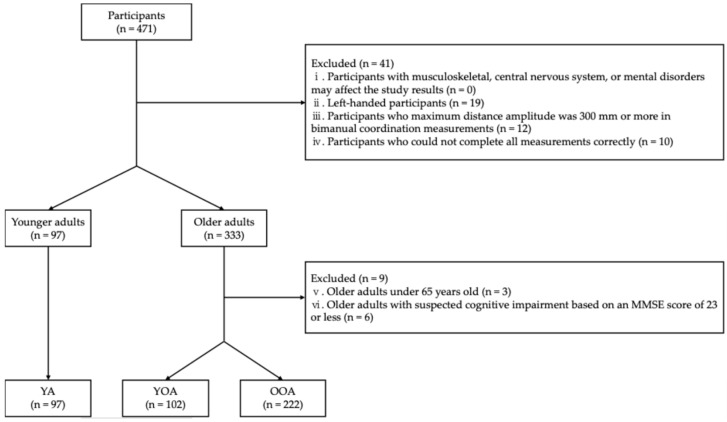
Flowchart of participation criteria. MMSE: Mini-mental state examination; YA: Young adult; YOA: Young-old adult; OOA: Old-old adult.

The study included 471 participants: 107 healthy younger adults and 364 healthy older adults. Younger adults were recruited from undergraduate students at K university, where they were affiliated, through the efforts of the collaborators H.N., S.S., and S.F. Their measurements were conducted at the university from December 2023 to February 2024. Older adults were recruited from community-dwelling individuals who participated in annual physical fitness testing sessions in the I and Y cities. Recruitment was facilitated through public information magazines issued by the cities’ public information and secretarial departments, as well as through local comprehensive support centers. Measurements for older adults took place at designated sites in September 2023. First, we set exclusion criteria (i)–(iv) for all participants. The exclusion criteria were as follows: (i) participants with musculoskeletal, central nervous system, or mental disorders that may have affected the study results; (ii) left-handed participants [16]; (iii) participants whose maximum amplitude of distance was 300 mm or more in bimanual coordination measurements [17]; (iv) participants who could not complete all measurements correctly. In this study, controlling for participants’ dominant hand was necessary to eliminate its influence on bimanual coordination. Therefore, left-handed participants, who comprised the smallest of the population, were excluded. Next, exclusion criteria (v) and (vi) were set for the older adult participants. The exclusion criteria were as follows: (v) older adults under 65 years; (vi) older adults with suspected cognitive impairment based on a Mini-Mental State Examination (MMSE) score of 23 or less [18]. After excluding the participants who met the exclusion criteria, participants were categorized into three groups: young adults (18–22 years), young-old adults (65–74 years), and old-old adults (≥75 years) [19] (Figure 1).

### 2.2. Ethics Statement

This study was conducted in accordance with the principles of the Declaration of Helsinki and approved by the Research Ethics Committee of Kyoto Tachibana University (approval number: 24-31, approval date: 22 July 2024). Informed consent was obtained from all the participants. The study was registered in the UMIN Clinical Trials Registry (UMIN000056499, register date: 22 July 2024).

### 2.3. Methods

**Figure 2:**
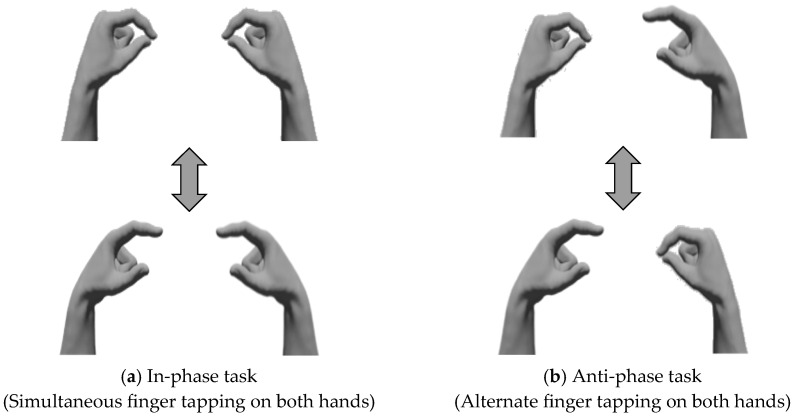
Bimanual coordination task. (**a**) In-phase task: Participants performed tapping movements of the thumb and index finger simultaneously on both sides. (**b**) Anti-phase task: Participants performed tapping movements of the thumb and index finger alternately on both sides.

**Figure 3:**
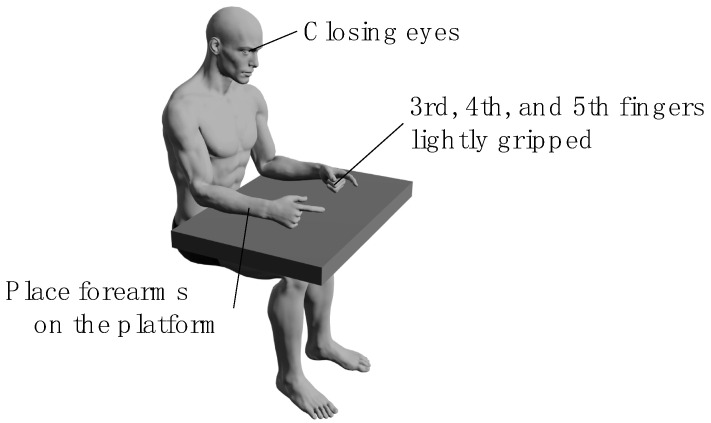
Measurement position of the bimanual coordination task. Participants sat on chairs with backrests and placed their forearms on the platform. During each task, the forearms were positioned in a neutral rotation, with the third, fourth, and fifth fingers slightly flexed, and measurements were taken with the participants sitting with eyes closed.

All the participants performed a bimanual coordination task involving opposition movements of the thumb and index finger [16] in two tasks: the in-phase task, where tapping movements of the thumb and index finger were performed simultaneously and as quickly as possible with both hands; and the anti-phase task, where tapping movements were alternated between the left and right hands [20] (Figure 2), wherein participants sat on chairs with backrests and placed their forearms on the platform. During each task, the forearms were positioned in a neutral rotation, with the third, fourth, and fifth fingers slightly flexed, and measurements were taken with the eyes closed (Figure 3). We instructed the participants to “perform as fast as possible and in the same rhythm.” The tasks were performed in sequence, starting with the in-phase task and followed by the anti-phase task. Each task was conducted for 15 s, and a 15-s pre-practice session was provided before each task.

Bimanual coordination performance was measured using a magnetic sensor finger-tapping device (UB-2, Maxell Ltd. Tokyo, Japan) [16]. Magnetic sensors were attached to the dorsal side of the participant’s thumb and index finger with a rubber band to measure the distance based on the strength of the magnetic field generated between the two fingers. This device was highly reproducible and reliable across periods, devices, and measurement examiners [21]. In this study, the participants were instructed to open their fingers to a width of 40 mm to minimize amplitude variation, and both pre-practice sessions and measurements were conducted [22]. Measurements for both younger and older adults were performed by a total of seven physiotherapists (including Y.S., S.M., H.N., S.S., and others) who were experienced in operating the UB-2 device. The features of bimanual coordination were obtained from the recorded data [20] (Table 1), which yielded four parameters for evaluating the distance and movement amplitude of the thumb and index finger during the task. Tap-interval-related parameters yielded four parameters for evaluating the average speed of movement and variability of tapping. The phase difference-related parameters yielded one parameter to evaluate the timing discrepancy of tapping between the hands. For each parameter, larger values for the total traveling distance, average local maximum distance, number of taps, and frequency of taps indicate better bimanual coordination performance. Conversely, smaller values for the standard deviation of the local maximum distance, slope of the approximate line of local maximum points, average of tapping interval, standard deviation of the inter-tapping interval, and standard deviation of phase difference indicate better performance for bimanual coordination. We instructed the participants to open their fingers to a width of 40 mm. Therefore, the closer the mean of the average of the local maximum distance is to 40 mm, the better the performance of bimanual coordination.

### 2.4. Statistical Analysis

First, a chi-square test was conducted to compare the male/female ratios among the young, young-old, and old-old adults. Next, a three-way analysis of variance (ANOVA) with a mixed design was conducted to compare the parameters related to the distance and tap interval, considering the factors of the hand (left, right), task (in-phase task, anti-phase task), and group (young, young-old, and old-old adults). A two-way mixed-design ANOVA was used to compare the parameters related to the phase difference considering task (in-phase task, anti-phase task) and group (young, young-old, and old-old adults) factors. Bonferroni post-hoc tests were performed for parameters with significant interactions or main effects using ANOVA. Furthermore, Pearson correlation analysis was conducted to examine the relationship between age and bimanual coordination performance. In addition, receiver operating characteristic (ROC) curves were generated to calculate the age cut-off points for bimanual coordination for the parameters that were significantly different among all three groups (young adult, young-old adult, and old-old adult) as a result of the post-hoc tests. The ROC curve was used to calculate the area under the curve (AUC), as well as sensitivity and specificity. In general, the closer the AUC is to 1, the better the overall diagnostic performance of the test, and the closer it is to 0.5, the poorer the test performance. Youden’s index was used to determine the age cut-off points for bimanual coordination for each parameter, calculated as the maximum value of {sensitivity + specificity − 1} [23]. SPSS version 29.0 (IBM Corp., Armonk, NY, USA) was used for the statistical analysis, with the significance level set at 5%.

## 3. Results

### 3.1. Basic Information of Participants

The 421 participants were divided into groups based on age: 97 young adults (male: 25, female: 72, age: 20.73 ± 1.43 years), 102 young-old adults (male: 17, female: 85, age: 70.77 ± 2.70 years), and 222 old-old adults (male: 52, female: 170, age: 80.71 ± 4.38 years). The results of the chi-square test showed no significant differences between male and female ratios among the young, young-old, and old-old adult groups (*p* > 0.05).

### 3.2. Comparison of the Distance Parameters

The statistical analysis revealed no significant three-way interaction (hand × task × group) for any distance parameter (*p* > 0.05; Table 2). The total traveling distance showed significant interactions between the hand × group and task × group factors (*p* < 0.05), and the total traveling distance had a significant effect on the task factor (*p* < 0.05). Post-hoc test results indicated that the total traveling distance in the old-old adult group was significantly lower for the left hand than for the right hand (*p* < 0.05). Moreover, the total traveling distance was significantly greater during the in-phase task than during the anti-phase task for the young, young-old, and old-old adult groups (*p* < 0.05). The total traveling distance in the anti-phase task was significantly lower in the old-old adult group than in the young and young-old adult groups (*p* < 0.05).

The average local maximum distance showed a significant interaction for the task × group factor (*p* < 0.05). The average local maximum distance showed significant main effects for both the task and group factors (*p* < 0.05). The results of post-hoc tests indicated that the average local maximum distance was significantly greater during the anti-phase task than during the in-phase task for the young, young-old, and old-old adult groups (*p* < 0.05). Furthermore, the average local maximum distance was significantly higher in the young-old and old-old groups than in the young adult group during the in-phase and anti-phase tasks (*p* < 0.05).

The standard deviation of the local maximum distance showed significant interactions for both hand × group and task × group factors (*p* < 0.05). Moreover, the standard deviation of the local maximum distance showed significant main effects for hand, task, and group factors (*p* < 0.05). The results of post-hoc tests indicated that the standard deviation of the local maximum distance was significantly higher for the left hand than for the right hand in the young, young-old, and old-old adult groups (*p* < 0.05), and the standard deviations were significantly greater in the anti-phase task than in the in-phase task (*p* < 0.05). Furthermore, the standard deviation of the local maximum distance for the right hand was significantly higher in the young-old and old-old adult groups than in the young adult group (*p* < 0.05). The standard deviation of the local maximum distance in the anti-phase task was significantly higher in the old-old adult group than in the young adult group (*p* < 0.05).

The slope of the approximate line of the local maximum points showed a significant interaction for the hand × task factor (*p* < 0.05). Additionally, the slope of the approximate line of the local maximum points showed significant main effects for both hand and task factors (*p* < 0.05). Post-hoc tests indicated that the slope of the approximate line of local maximum points in the in-phase task for the left hand was significantly lower than that for the right hand (*p* < 0.05). Furthermore, the slope of the approximate line of the local maximum points for the right hand was significantly lower in the anti-phase task than that in the in-phase task (*p* < 0.05).

### 3.3. Comparison of the Tap Interval Parameters

Statistical analysis revealed no significant three-way interaction (hand × task × group) for any of the tap-interval parameters (*p* > 0.05; Table 2).

**Figure 4:**
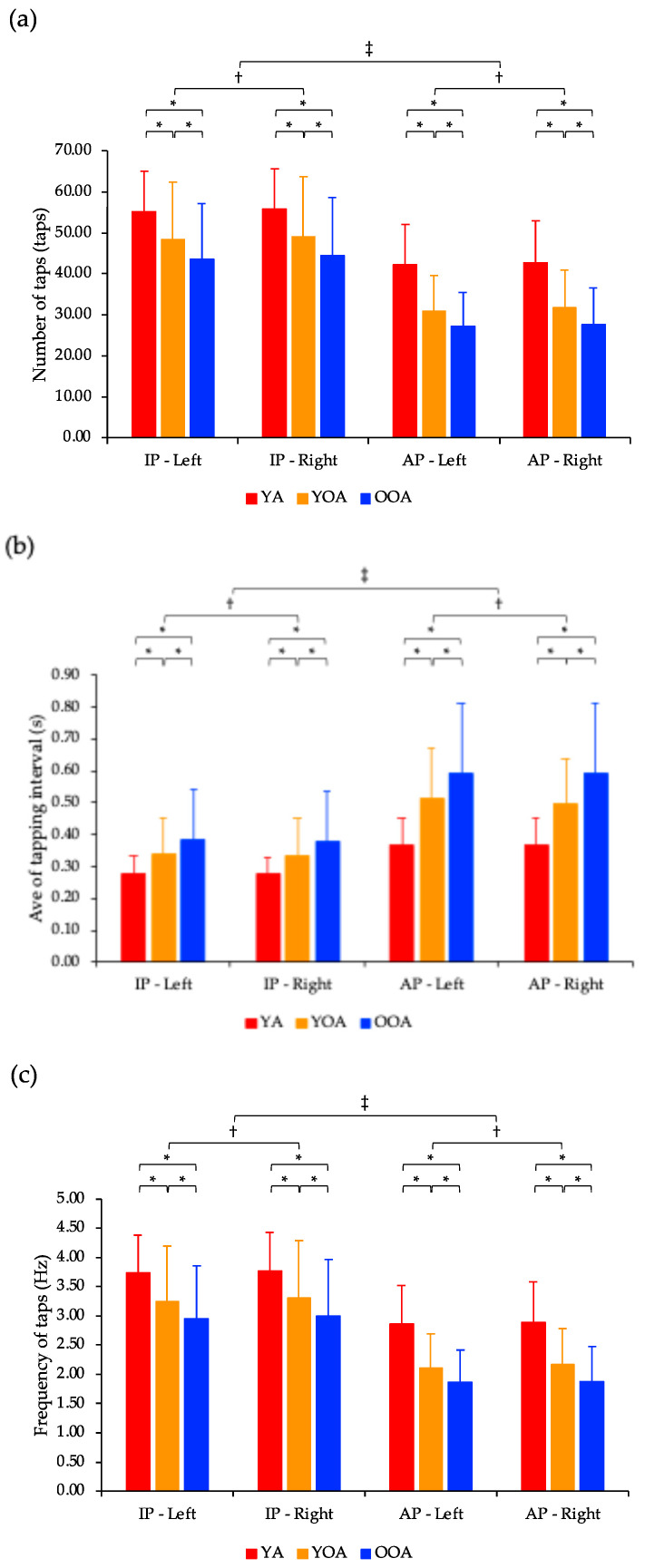
Comparison of in-phase and anti-phase tasks and left and right hands based on the number of taps, average of tapping interval, and frequency of taps. (**a**) The number of taps showed a significant interaction effect between task and group factors (*p* < 0.05). The number of taps showed significant main effects for the hand, task, and group factors (*p* < 0.05). Post-hoc tests indicated that the number of taps in both the in-phase and anti-phase tasks decreased significantly in the following order: young adults, young-old adults, and old-old adults (* *p* < 0.05). Furthermore, the number of taps was significantly lower for the left hand than for the right hand (^†^ *p* < 0.05). Finally, the number of taps in the young, young-old, and old-old adult groups was significantly lower in the anti-phase task than in the in-phase task (^‡^ *p* < 0.05). (b) The average of tapping interval showed a significant interaction effect between the task and group factors (*p* < 0.05). Additionally, the average of tapping interval showed significant main effects for hand, task, and group factors (*p* < 0.05). Post-hoc test results indicated that the average of tapping interval in both the in-phase and anti-phase tasks significantly increased in the following order: young adults, young-old adults, and old-old adults (* *p* < 0.05). Furthermore, the average of tapping interval was significantly longer for the left hand than for the right hand (^†^ *p* < 0.05). Finally, the average of tapping interval in the young, young-old, and old-old adult groups were significantly longer in the anti-phase task than in the in-phase task (^‡^ *p* < 0.05). (c) The frequency of taps showed a significant interaction effect between task and group factors (*p* < 0.05). Additionally, the frequency of taps showed significant main effects for hand, task, and group factors (*p* < 0.05). Post-hoc tests indicated that the frequency of taps in both the in-phase and anti-phase tasks decreased significantly in the following order: young adults, young-old adults, and old-old adults (* *p* < 0.05). Furthermore, the frequency of taps was significantly lower in the left hand than in the right hand (^†^ *p* < 0.05). Finally, the frequency of taps in the young, young-old, and old-old adult groups was significantly lower in the anti-phase task than in the in-phase task (^‡^ *p* < 0.05). Ave: Average; S: Second; IP: In-phase task; AP: Anti-phase task; YA: Young adult; YOA: Young-old adult; OOA: Old-old adult.

The number of taps showed a significant interaction effect between task and group factors (*p* < 0.05; Figure 4a). The number of taps showed significant main effects for the hand, task, and group factors (*p* < 0.05). Post-hoc tests indicated that the number of taps was significantly lower for the left hand than for the right hand (*p* < 0.05). Furthermore, the number of taps in the young, young-old, and old-old adult groups was significantly lower in the anti-phase task than in the in-phase task (*p* < 0.05). Finally, the number of taps in both the in-phase and anti-phase tasks decreased significantly in the following order: young adults, young-old adults, and old-old adults (*p* < 0.05).

The average of tapping interval showed a significant interaction effect between the task and group factors (*p* < 0.05; Figure 4b). Additionally, the average of tapping interval showed significant main effects for hand, task, and group factors (*p* < 0.05). Post-hoc test results indicated that the average of tapping interval was significantly longer for the left hand than for the right hand (*p* < 0.05). Moreover, the average of tapping interval in the young, young-old, and old-old adult groups were significantly longer in the anti-phase task than in the in-phase task (*p* < 0.05). Finally, the average of tapping interval in both the in-phase and anti-phase tasks significantly increased in the following order: young adults, young-old adults, and old-old adults (*p* < 0.05).

The frequency of taps showed a significant interaction effect between task and group factors (*p* < 0.05; Figure 4c). Additionally, the frequency of taps showed significant main effects for hand, task, and group factors (*p* < 0.05). Post-hoc tests indicated that the frequency of taps was significantly lower in the left hand than in the right hand (*p* < 0.05). Furthermore, the frequency of taps in the young, young-old, and old-old adult groups was significantly lower in the anti-phase task than in the in-phase task (*p* < 0.05). Finally, the frequency of taps in both the in-phase and anti-phase tasks decreased significantly in the following order: young adults, young-old adults, and old-old adults (*p* < 0.05).

The standard deviation of inter-tapping interval showed significant interaction effects for the task × group and hand × task factors (*p* < 0.05). Additionally, the standard deviation of inter-tapping interval showed significant main effects for hand, task, and group factors (*p* < 0.05). Post-hoc test results indicated that the standard deviation of inter- tapping interval was significantly higher for the left hand than for the right hand in both the in-phase and anti-phase tasks (*p* < 0.05). Moreover, the standard deviation of inter- tapping interval in the young-old and old-old groups was significantly higher in the anti-phase task than in the in-phase task for both hands (*p* < 0.05). Finally, the standard deviation of inter-tapping interval in the in-phase task was higher in the young-old adult group and the old-old adult group than in the young adult group, whereas it significantly increased in the order of young adults, young-old adults, and old-old adults in the anti-phase task (*p* < 0.05).

### 3.4. Comparison of the Phase Difference Parameters

The standard deviation of phase difference showed a significant interaction effect for the task × group (*p* < 0.05). Additionally, the standard deviation of phase difference showed significant main effects for both task and group factors (*p* < 0.05). Post-hoc test results indicated that the standard deviation of phase difference for the young-old and old-old adult groups was significantly higher in the anti-phase task than in the in-phase task (*p* < 0.05). The standard deviation of phase difference in the anti-phase task increased significantly in the following order: young adults, young-old adults, and old-old adults (*p* < 0.05; Table 3).

### 3.5. Correlation Analysis Between the Bimanual Coordination Performance and Age

The results of the correlation analysis indicated that the average local maximum distance, number of taps, average of tapping interval, frequency of taps, standard deviation of inter-tapping interval in the in-phase and anti-phase tasks, total traveling distance of the left hand, standard deviation of the local maximum distance, and standard deviation of phase difference in the anti-phase task were strongly correlated with age; however, the correlations were very weak. The only moderate correlation with age was observed in the number of taps and frequency of taps in the anti-phase task (*p* < 0.05; Table 4).

### 3.6. Age Cut-Off Points for Bimanual Coordination Performance

**Figure 5:**
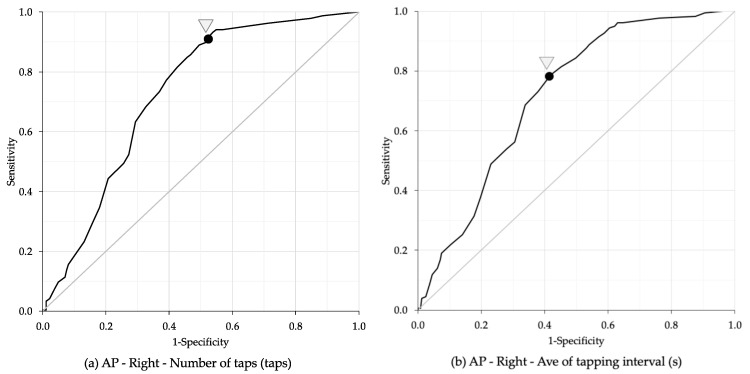

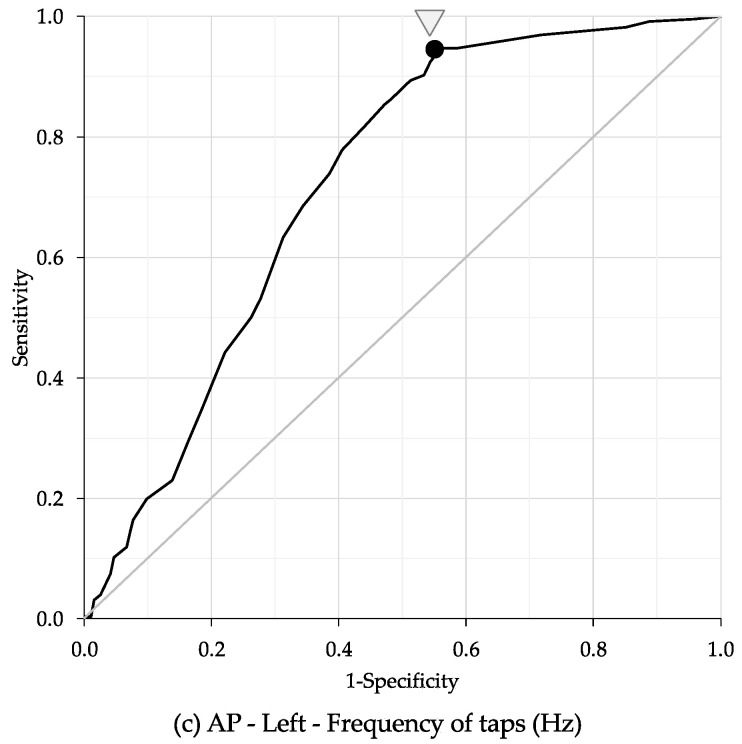
Receiver operating characteristic (ROC) analysis between bimanual coordination task and age. (**a**) Number of taps: the highest values were observed in the right hand during the anti-phase task, with an AUC of 0.73, sensitivity of 89.0%, specificity of 50.5%, and an age cut-off point of 68.5 years. (**b**) Average of tapping interval: the highest values were observed in the right hand during the anti-phase task, with an AUC of 0.72, sensitivity of 78.7%, specificity of 58.0%, and an age cut-off point of 73.5 years. (**c**) Frequency of taps: the highest value was observed in the left hand of the anti-phase task, with an AUC of 0.72, sensitivity of 93.8%, specificity of 44.6%, and an age cut-off point of 65.5 years. Ave: Average; S: Second; IP: In-phase task; AP: Anti-phase task.

The ROC curve analysis identified age cut-off points for bimanual coordination performance based on the number of taps, average of tapping interval, and frequency of taps (Table 5). The highest AUC for the number of taps was observed in the right hand during the anti-phase task, with an AUC of 0.73, sensitivity of 89.0%, specificity of 50.5%, and an age cut-off point of 68.5 years (Figure 5a). The highest AUC for average of tapping interval was also found in the right hand during the anti-phase task, with an AUC of 0.72, sensitivity of 78.7%, specificity of 58.0%, and an age cut-off point of 73.5 years (Figure 5b). The highest AUC for frequency of taps was recorded in the left hand during the anti-phase task, with an AUC of 0.72, sensitivity of 93.8%, specificity of 44.6%, and an age cut-off point of 65.5 years (Figure 5c).

## 4. Discussion

This study aimed to clarify age-related changes in bimanual coordination by comparing the performance of young, young-old, and old-old adults on bimanual coordination tasks. The results showed that the performance on the bimanual coordination task was lower in the older adult group than in the young adult group. In particular, the number and frequency of taps decreased, while the average of tapping interval increased in the following order: young adults, young-old adults, and old-old adults. Conversely, the average local maximum distance and the standard deviation of the local maximum distance increased in the older adult group than in the young adult group but remained consistent between the young-old and old-old adult groups. Furthermore, the performance of the anti-phase task was lower than that of the in-phase task, and the left-hand performance was lower than that of the right hand. Furthermore, age cut-off values for bimanual coordination were identified for the number of taps, average of tapping interval, and frequency of taps. These results suggest that the number of taps, average of tapping interval, and frequency of taps are potential tools for assessing age-related changes in bimanual coordination. Additionally, the measurement results may help individuals assess whether their performance is superior or inferior compared to others in the same age group. This information could facilitate early detection of bimanual coordination decline and support preventive measures at the preliminary stage for each age group.

### 4.1. Comparison of Young Adults, Young-Old Adults, and Old-Old Adults

In this study, the performance of bimanual coordination tasks was compared among young, young-old, and old-old adult groups. The parameters were categorized into those that changed with aging among the young adult, young-old adult, and old-old adult groups, and those that showed differences between the young adult and older adult groups but no difference between the young-old adult and old-old adult groups.

The number and frequency of taps decreased in the order of young adult, young-old adult, and old-old adult groups during both in-phase and anti-phase tasks. Conversely, the average of tapping interval increased in the order of young adult, young-old adult, and old-old adult groups during both the in-phase and anti-phase tasks. This indicates that aging is associated with a reduction in the number of taps and an increase in the time required for a single-tapping action. A study comparing bimanual performance between young and young-old adults reported that the young-old adult group exhibited a reduction in the number of finger taps and an extension of movement time, leading to a decline in task performance compared to the young adult group [11]. Furthermore, in a bimanual force regulation task, aging was found to increase performance errors, leading to a decline in the ability to control coordinated forces with both hands in the young, young-old, and old-old adult groups [24]. In summary, aging appears to impair bimanual coordination and reduce both movement quantity and accuracy. Consequently, it is possible that, in this study, the number of taps decreased, and the time required for a single-tapping action increased in the order of young adults, young-old adults, and old-old adults. Additionally, interhemispheric interactions via the corpus callosum play a crucial role in bimanual coordination [25], and age-related structural and functional changes in the corpus callosum have been reported to impair bimanual performance [26,27]. These findings suggest that the performance of bimanual coordination tasks declined sequentially from the young adult group to the young-old adult group to the old-old adult group, potentially because of age-related impairments in the interaction between the left and right cerebral hemispheres.

The standard deviation of inter-tapping interval increased during the anti-phase task in the following order: young adult, young-old adult, and old-old adult. This indicates that the variability in the time required for a single tapping action during an anti-phase task increases with age. Previous studies examining age-related changes in bimanual force control during in- and anti-phase tasks have reported greater variability in the time taken to exert the specified force among older adults compared with young adults, with a particularly pronounced decline in performance during anti-phase tasks compared with in-phase tasks [12]. Furthermore, age-related impairments in neuromodulation in the brain can lead to random activation of neurons, resulting in increased intraindividual performance variability [28]. Therefore, in addition to changes in motor performance due to task difficulty, increased variability in motor performance resulting from age-related impairments in neural regulation within the brain led to a progressive increase in the standard deviation of inter-tapping interval from the young adult group to the young-old adult group and from the young-old adult group to the old-old adult group. Similarly, the standard deviation of phase differences during the anti-phase task increased in the order of the young adult, young-old adult, and old-old adult groups. This indicated that the timing discrepancy between the tapping movements of the right and left hands increased with age. The accuracy of bimanual coordination declines in older adults compared with young adults [9]. Additionally, a study on nerve conduction velocity in the peripheral nervous system, spanning ages 20 to 103 years, revealed a linear decrease in the average conduction velocity with age in both males and females [29]. In this study, it is possible that the peripheral nerve conduction velocity declined with age. Consequently, the timing discrepancy in tapping movements between the right and left hands likely increased in the order of the young adult, young-old adult, and old-old adult groups. Furthermore, rhythmic bimanual coordination stability is influenced by neural crosstalk, movement amplitude, and conduction time delays [30]. Therefore, peripheral nerve conduction velocity may have declined with age in this study. In summary, the age-related decline in peripheral nerve conduction velocity may have impaired rhythmic bimanual coordination, resulting in a progressive increase in the standard deviation of phase difference from the young adult group to the young-old adult group and from the young-old adult group to the old-old adult group. In this study, differences between the groups in the standard deviations of inter-tapping interval and phase differences were observed only during the anti-phase task. The supplementary motor area plays a crucial role in the bilateral motor control during bimanual coordination. Previous studies have shown that in healthy young adults, activity in the supplementary motor area is more prominent during anti-phase movements than during in-phase movements. However, motor facilitation connectivity within cortical motor networks, including the supplementary motor area, decreases with age [31,32]. These findings suggest that decreased activity of the supplementary motor area involved in anti-phase movements due to aging may have caused the observed differences in the variability of single-tap movements and inter-hand coordination, specifically in the anti-phase task, among young adults, young adults, and old-old adults.

The average local maximum distance significantly increased in the young-old and old-old adult groups compared to the young adult group during both the in-phase and anti-phase tasks. This indicates that the opening width between the thumb and index finger increased in the older adult group compared to the young adult group, regardless of task difficulty. Motor control in bimanual coordination declines with age, and accuracy decreases in older adults compared to young adults [9]. Additionally, working memory, which is the ability to temporarily store and actively process information to achieve specific goals, has been reported to decline with age [33]. In the bimanual coordination task used in this study, the participants were instructed to maintain a finger separation width of 40 mm between the thumb and index finger and were required to perform the tasks while preserving this interval. Therefore, in this study, the decline in working memory due to aging may have prevented participants from maintaining a consistent distance between the thumb and index finger, leading to an increase in the average local maximum distance in the older adult group compared with the young adult group.

The standard deviation of the local maximum distance increased in the right hand for the young-old and old-old adult groups compared to the young adult group. This indicated that the variability in the opening width of the fingers was greater in the older adult group than in the young adult group for the right hand. Bimanual coordination variability has been reported to be higher in older adults than in young adults [9]. Moreover, proprioception in the fingers declines with age. Older adults show longer proprioceptive reaction times and reduced position sense than younger adults [34]. It is possible that the age-related decline in position sense made it difficult for older adults to consistently maintain the opening width of their fingers. Thus, the variability in finger-opening width during the bimanual coordination task may have been greater in the older adult group than in the younger adult group. Additionally, studies on unilateral and bilateral upper limb targeting tasks have shown that the task accuracy is generally higher for the right hand than for the left hand [35]. Therefore, the variability in the opening width of the fingers during bimanual coordination tasks may have been reduced in the dominant right hand compared to the non-dominant left hand. These findings suggest that the standard deviation of the local maximum distance was larger in the older adult group than in the young adult group for the right hand.

The standard deviation of inter-tapping interval increased in the in-phase task for the young-old and old-old adult groups compared with the young adult group. This indicates that the variability in the time required for a single-tapping action was greater in the older adult group than in the young adult group during the in-phase task. Previous studies have reported that motor performance in bimanual coordination declines significantly in older adults compared to young adults [11,12]. Additionally, older adults exhibit a trade-off between movement frequency and motor control accuracy. They tended to compensate for the reduced accuracy by decreasing the frequency of bimanual coordination movements [11]. In this study, frequency of taps, which represents the frequency of single-tapping actions, decreased in the following order: young adults, young-old adults, and old-old adults. However, the standard deviation of inter-tapping interval, representing variability in the time required for a single-tapping action, increased in the older adult group compared with that in the young adult group. Moreover, in this study, the slope of the approximate line of the local maximum points, which reflects fatigue, decreased more during the anti-phase task than during the in-phase task. This suggests that fatigue develops more easily in the in-phase task than in the antiphase task. Additionally, the number of taps performed by the older adult group was higher in the in-phase than in the anti-phase task. Thus, it is possible that in older adults, the increased number of taps during the in-phase task compared to the anti-phase task led to greater fatigue, which in turn resulted in increased variability in the time required for a single-tapping action. These findings suggest that the standard deviation of inter-tapping interval may have been higher in older adults than in young adults during in-phase tasks.

In summary, this study demonstrated that the performance on bimanual coordination tasks declines in older adults compared to young adults. The average of local max distance and standard deviation of local maximum distance, which reflect the distance of the bimanual coordination task, were greater in the older adult group than in the young adult group. Furthermore, among older adults, the number and frequency of taps decreased in the following order: young, young-old, and old-old. Conversely, the average of tapping interval increased in the following order: young adults, young-old adults, and old-old adults. These findings suggest that the number of taps, average of tapping interval, and the frequency of taps, which reflect the speed of bimanual coordination, could serve as useful indicators for assessing age-related changes in bimanual coordination performance among young, young-old, and old-old adults.

### 4.2. Comparison of the In-Phase and the Anti-Phase Tasks

In this study, the total traveling distance, number of taps, and frequency of taps decreased during the anti-phase task compared to the in-phase task among young, young-old, and old-old adults. Conversely, the average local maximum distance and the average of tapping interval increased during the anti-phase task compared with the in-phase task among young adults, young-old adults, and old-old adults. This indicates that, regardless of the participants’ age, the amount of hand movement and the number of taps decreased during the anti-phase task compared to the in-phase task, whereas the time required for a single tap and the range of hand opening increased during the anti-phase task compared to the in-phase task. In the in-phase task, the tapping movements of the thumb and index finger were performed with both hands, and in the anti-phase task, the tapping movements were alternated between the left and right hands. The anti-phase task requires different movements of the left and right hands, making it more challenging than the in-phase task. Consequently, bimanual coordination performance is expected to decline in the anti-phase task compared to the in-phase task. It has been shown that bimanual motor impairments increase during asymmetrical bimanual tasks like the anti-phase task compared to symmetrical tasks like the in-phase task, and these impairments are more pronounced in older adults than in young adults [10]. Research on behavioral principles in inter-limb and hand-foot coordination has reported that during anti-phase movements, interference from one hand’s movement affects the other hand’s movement, resulting in spatial and temporal constraints. As the frequency of anti-phase movements increases, there is a tendency to shift toward in-phase movements for symmetry [36]. In other words, in the in-phase task, the movements of one hand likely facilitated the movements of the other hand, enhancing performance in the bimanual coordination task. In contrast, in the anti-phase task, the movements of one hand may interfere with those of the other, leading to reduced performance in the bimanual coordination task. Indeed, in this study, the number and frequency of taps increased in the less demanding in-phase task and decreased in the more demanding anti-phase task. Thus, the performance of bimanual coordination tasks appears to depend on task difficulty. This suggests that both the young adult and older adult groups in this study likely experienced a decline in performance during the anti-phase task compared to the in-phase task. Furthermore, it has also been reported that in young adults, the greater the microstructural integrity in the midbrain regions, the better the motor performance in tasks requiring interactions between the cerebral hemispheres [37]. Additionally, compared with young adults, older adults tend to have smaller anterior fibers of the corpus callosum and poorer performance on anti-phase bimanual coordination tasks [38]. These findings suggest that in addition to task difficulty, the reduced amount of movement and number of taps observed during the anti-phase task in this study may be influenced by the structural integrity of the corpus callosum fibers in young and older adults, particularly in tasks requiring alternating bimanual coordination.

Furthermore, in this study, the standard deviation of inter-tapping interval increased during the anti-phase task compared with the in-phase task for both the left and right hands. This indicates that the variability in the time required for a single-tapping movement was greater in the anti-phase task than in the in-phase task. It has been reported that the performance of bimanual coordination task—such as regarding accuracy, variability, and execution time—decreases during asymmetrical tasks compared to symmetrical tasks [16,38,39]. Asymmetrical tasks are generally considered more challenging than symmetrical tasks. The standard deviation of inter-tapping interval, which is one of the parameters in this study, serves as an indicator of variability in bimanual coordination tasks. An in-phase task corresponds to a symmetrical task, whereas an anti-phase task corresponds to an asymmetrical task. These findings suggest that temporal variability in the time required for a single-tapping motion increased in the anti-phase task compared to the in-phase task because of the influence of task difficulty.

In addition, the standard deviations of the local maximum distance, inter-tapping interval, and phase difference increased during the anti-phase task compared to the in-phase task in both the young-old and old-old adult groups. This indicates that, in the older adult group, the variability in the range of hand opening, time required for a single-tapping movement, and timing discrepancies between the hands were greater during the anti-phase task than during the in-phase task. It has been reported that the variability in bimanual coordination increases with age, with older adults exhibiting more pronounced differences between in-phase and anti-phase tasks than younger adults. While no differences in the performance of bimanual coordination tasks were observed between the two tasks in young adults, older adults tended to experience reduced performance on the bimanual coordination task during the anti-phase task compared to the in-phase task [38]. This suggests that the reduction in corpus callosum size and the decline in its microstructural integrity with aging may have led to an increase in the standard deviation of local maximum distance, standard deviation of inter-tapping interval, and standard deviation of phase difference in the older adult group during the anti-phase task compared with the in-phase task, but not in the young adult group. This indicates that older adults’ performance on bimanual coordination tasks tends to be more dependent on task difficulty than younger adults. It is also more prone to decline during high-difficulty antiphase tasks, which require greater interhemispheric interactions.

Conversely, the slope of the approximate line of the local maximum points decreased in the right hand during the anti-phase task compared to the in-phase task. This indicated that the anti-phase task was less influenced by fatigue than the in-phase task. Indeed, in this study, the number and frequency of taps decreased, while the average of tapping interval increased during the anti-phase task compared to the in-phase task. In other words, the in-phase task involved shorter average of tapping interval and a higher number of taps than the anti-phase task, which may have made it more prone to fatigue. A study comparing maximal grip strength and endurance between the dominant and nondominant hands reported that while the dominant hand exhibited greater absolute strength, it was more susceptible to early fatigue [40]. Furthermore, it has been noted that the muscle fiber composition in the fingers of the dominant hand includes a significantly higher proportion of Type II fibers, which excel in rapid force generation, compared to Type I fibers, which are better suited for endurance [41]. These findings suggest that the reduced endurance of the dominant right hand resulted in a decreased slope of the approximate line of local maximum points during the anti-phase task compared to the in-phase task.

In summary, this study revealed that the performance on bimanual coordination tasks declined during the anti-phase task compared to the in-phase task. The number of bimanual coordination tasks decreased during the anti-phase task among all age groups, including young, young-old, and old-old adults. In contrast, spatial and temporal variability in bimanual coordination tasks was greater in the anti-phase task than in the in-phase task only in the older adult group. These findings suggest that the performance of bimanual coordination tasks may decrease during the anti-phase task compared to the in-phase task among young, young-old, and old-old adults. Furthermore, spatial and temporal variability in bimanual coordination tasks tended to increase more prominently in young-old and old-old adults than in young adults.

### 4.3. Comparison of the Non-Dominant Hand (Left Hand) and Dominant Hand (Right Hand)

In this study, the total traveling distance, number of taps, and frequency of taps were lower for the left hand than for the right hand. However, the standard deviation of the local maximum distance, average of tapping interval, and standard deviation of inter-tapping interval were higher for the left hand than for the right hand. This indicates that the left hand has less finger movement compared to the right hand and shows greater variability in the distance between the thumb and index finger, as well as in the time required for each tapping motion compared to the right hand. Repetitive movement tasks may have been suggested to induce changes in activity within the motor cortex, a phenomenon known as use-dependent plasticity [42]. Research on handgrip strength and finger dexterity between the dominant and non-dominant hands has reported significantly stronger grip strength and higher finger dexterity in the dominant hand than in the non-dominant hand [43]. Notably, all the participants in this study were right-handed, as left-handed individuals were excluded based on previous studies [16]. Therefore, it is possible that use-dependent plasticity resulted in lower performance in the left hand than in the right hand across many parameters in the bimanual coordination task. Furthermore, the total traveling distance was lower in the left hand than in the right hand in the old-old adult group. This indicates that finger movement in the old-old adult group was lower in the left hand than in the right hand. It has been reported that finger usage of the dominant hand increases with age compared with that of the non-dominant hand [44]. Therefore, age-related increases in finger usage might have caused a left-right difference in finger movement in the old-old adult group, the oldest age group. These findings suggest that bimanual coordination performance may be lower in the older adult group than in the young adult group and lower in the non-dominant left hand than in the dominant right hand.

Next, the standard deviation of inter-tapping interval was higher for the left hand than for the right hand for both the in-phase and anti-phase tasks. This indicated that the variability in the time required for a single tapping movement was greater in the left hand, regardless of the task type. In general, right-handed individuals exhibit higher manual dexterity in their dominant hand than in their non-dominant hand [44]. Therefore, the standard deviation of inter-tapping interval, which represents temporal variability, increased in the non-dominant left hand compared with the dominant right hand owing to use-dependent plasticity.

Furthermore, the standard deviation of the local maximum distance was higher for the left hand than for the right hand across all age groups, including young, young-old, and old-old adults. This indicated that the variability in the distance required for a single tapping movement was greater in the left hand than in the right hand, regardless of age. A previous study that used tasks that required drawing in-phase and anti-phase circles with both hands reported that movement variability was lower in the dominant hand than in the non-dominant hand [45]. Therefore, the standard deviation of the local maximum distance, which represents spatial variability, is thought to have increased in the non-dominant left hand compared to the dominant right hand.

The slope of the approximate line of the local maximum points during the in-phase task was lower for the left than for the right hand. This indicated that the left hand experienced less fatigue than the right hand during the in-phase task. The in-phase task was considered less challenging than the anti-phase task, and the increased movement during the task may have led to fatigue. A previous study reported that during tasks requiring simultaneous maximal force exertion with both hands, the dominant hand demonstrated a faster decline in force than the non-dominant hand [46]. In this study, the number and frequency of taps were lower in the left hand than in the right hand and were also lower during the anti-phase task than during the in-phase task. Similarly, the average of tapping interval were higher in the left hand than in the right hand, and higher during the anti-phase task than during the in-phase task. These results suggest that the dominant right hand in the in-phase task may have been the most fatigued hand because it moved the most during the task.

In summary, it was revealed that the performance of bimanual coordination task was lower in the non-dominant left hand compared to the dominant right hand. Notably, unlike the young and young-old adult groups, the old-old adult group showed considerably less movement in the non-dominant left hand than in the dominant right hand during the bimanual coordination task. These findings suggest that the left-right difference in the amount of movement in bimanual coordination tasks may increase with age.

### 4.4. Limitations

This study had some limitations. First, age-related changes in bimanual coordination were examined only at the behavioral level, leaving the neural mechanisms in the brain unclear. Second, this study focused solely on age-related changes in bimanual coordination using tapping tasks, and its relationship with other physical and psychophysiological functions remains uncertain. Sex differences in bimanual coordination between boys and girls have been reported [17]. The present study focused on the age-specific characteristics of bimanual coordination and conducted a three-way ANOVA with hand, task, and group factors. The addition of a sex factor may complicate the statistical analysis and make it difficult to interpret the effect of age. Therefore, sex could not be included in the statistical analysis in this study. However, we cannot rule out the possibility that sex-specific differences in bimanual coordination may also exist in young and older adults as well as in children. Furthermore, grip strength, one of the indices of muscle strength, has been reported to decline with aging, leading to reduced bimanual coordination performance [8]. Therefore, age-related changes in bimanual coordination may result not only from neurological factors, such as impaired interhemispheric interaction, but also from myological factors associated with age-related decline in muscle strength. In fact, in recent years, bimanual coordination tasks have been increasingly used as screening tests for cognitive impairment, and it has been suggested that they may also be associated with other physical and psychological functions [47]. Therefore, future studies should aim to clarify the neural mechanisms involved in bimanual coordination and their relationship with other physical and psychophysiological functions using techniques such as transcranial magnetic stimulation and electroencephalography.

## 5. Conclusions

We clarified age-related changes in bimanual coordination by comparing the performance of bimanual coordination tasks among young, young-old, and old-old adults. The performance of bimanual coordination tasks was lower in the non-dominant left hand than in the dominant right hand during the more challenging anti-phase task compared to the less challenging in-phase task. Furthermore, the features of the bimanual coordination task revealed parameters that showed minimal variations between young-old and old-old adults as well as among parameters that exhibited significant aging-related changes. Specifically, the number and frequency of taps decreased, whereas the average of tapping interval increased in the following order: young, young-old, and old-old adults. Furthermore, age cut-off values for bimanual coordination were identified for the number of taps, average of tapping interval, and frequency of taps. These findings suggest that these variables may be useful as tools for assessing aging-related changes in bimanual coordination. Additionally, the measurement results may help individuals assess whether their performance is superior or inferior compared to others in the same age group. This information could facilitate early detection of bimanual coordination decline and support preventive measures at the preliminary stage for each age group.

## Figures and Tables

**Table 1:** Characteristics of the bimanual coordination task.

	Parameter	Description
Distance	Total travel distance (mm)	The sum of the distances moved by the thumb and index finger. The overall amount of movement.
	Ave of local max distance (mm)	Average amplitude of the distance waveform.
	SD of local max distance (mm)	Variation in the amplitude of the distance waveform.
	Slope of approximate line of local max points (mm/s)	The slope is a linear regression of the relationship between the maximum point of each tap and time. As the tap amplitude decreases due to fatigue, the slope increases in a negative direction. When there is no effect of fatigue, the slope is 0.
Tap interval	Number of taps (taps)	Number of taps during the measurement time.
	Ave of tapping interval (s)	Average in time difference between two consecutive taps.
	Frequency of taps (Hz)	Inverse to the mean of the tap interval.
	SD of inter-tapping interval (s)	Variations in time difference between two consecutive taps.
Phase difference	SD of phase difference (degree)	Assuming the interval between one tap is 360°, the time lag between the left and right hands is expressed as an angle. This parameter is the variation of its value.

Ave: Average; Max: Maximum; SD: Standard deviation; S: Second; Phase difference: phase difference between the left and right taps.

**Table 2:** Comparison of in-phase and anti-phase tasks and left and right hands at distance and tap interval.

	Task	Hand	YA (*n* = 97)	YOA (*n* = 102)	OOA (*n* = 222)	IE	IE	IE	IE	ME	ME	ME	Post-Hoc Test		
						Hand × Group	Task × Group	Hand × Task	Hand × Group × Task	Hand	Task	Group			
						F	F	F	F	F	F	F	Hand	Task	Group
Total traveling distance (mm)	IP	L	4323.67	4498.38	4178.93	3.31 *	5.36 **	0.22	0.09	0.90	127.97 **	2.78	OOA: L < R ^a^	YA, YOA, OOA: AP < IP ^a^	AP: OOA < YOA, YA ^a^
			(1400.02)	(1581.46)	(1591.60)										
		R	4242.82	4568.94	4332.58										
			(1210.37)	(1510.33)	(1475.50)										
	AP	L	4001.68	3845.86	3471.61										
			(1297.70)	(1226.17)	(1165.46)										
		R	3883.48	3923.39	3595.16										
			(1191.90)	(1292.31)	(1221.34)										
Ave of local max distance (mm)	IP	L	41.07	46.81	48.11	1.71	10.10 **	1.25	0.82	0.32	354.23 **	24.23 **		YA, YOA, OOA: IP < AP ^a^	IP, AP: YA < YOA, OOA ^a^
			(12.34)	(16.04)	(17.03)										
		R	40.25	47.01	48.78										
			(10.65)	(14.23)	(16.07)										
	AP	L	49.50	60.55	62.22										
			(13.93)	(15.60)	(17.25)										
		R	47.39	59.90	63.15										
			(12.35)	(14.98)	(16.90)										
SD of local max distance (mm)	IP	L	6.95	6.71	6.63	5.18 **	8.50 **	2.96	1.13	98.92 **	44.38 **	3.91 *	YA, YOA, OOA: R < L ^a^	YOA, OOA: IP < AP ^a^	R: YA < YOA, OOA ^a^ AP: YA < OOA ^a^
			(2.57)	(2.27)	(2.23)										
		R	5.26	5.55	5.70										
			(1.62)	(2.08)	(2.03)										
	AP	L	7.06	7.36	7.90										
			(2.70)	(3.17)	(3.32)										
		R	5.45	6.87	7.09										
			(1.72)	(2.81)	(3.24)										
Slope of approximate line of local max points (mm/s)	IP	L	−0.14	−0.14	−0.09	1.23	0.45	4.03 *	1.70	10.56 **	6.82 **	0.63	IP: L < R ^a^	R: AP < IP ^a^	
			(0.59)	(0.66)	(0.67)										
		R	−0.08	0.11	0.03										
			(0.44)	(0.56)	(0.65)										
	AP	L	−0.21	−0.15	−0.15										
			(0.60)	(0.80)	(0.77)										
		R	−0.11	−0.10	−0.17										
			(0.49)	(0.67)	(0.72)										
Number of taps (taps)	IP	L	55.25	48.29	43.53	0.22	5.55 **	0.06	1.56	42.98 **	906.45 **	58.51 **	L < R ^b^	YA, YOA, OOA: AP < IP ^a^	IP, AP: OOA < YOA < YA ^a^
			(9.66)	(14.04)	(13.52)										
		R	55.74	48.95	44.42										
			(9.85)	(14.70)	(14.34)										
	AP	L	42.15	30.97	27.14										
			(9.86)	(8.75)	(8.40)										
		R	42.78	31.77	27.64										
			(10.27)	(9.19)	(8.83)										
Ave of tapping interval (s)	IP	L	0.28	0.34	0.38	1.48	20.41 **	2.92	2.40	15.75 **	370.25 **	49.93 **	R < L ^b^	YA, YOA, OOA: IP < AP ^a^	IP, AP: YA < YOA < OOA ^a^
			(0.05)	(0.11)	(0.16)										
		R	0.27	0.34	0.38										
			(0.05)	(0.12)	(0.16)										
	AP	L	0.37	0.51	0.59										
			(0.09)	(0.16)	(0.22)										
		R	0.37	0.50	0.59										
			(0.09)	(0.14)	(0.22)										
Frequency of taps (Hz)	IP	L	3.73	3.25	2.95	0.49	5.08 **	0.67	1.34	39.06 **	896.61 **	57.89 **	L < R ^b^	YA, YOA, OOA: AP < IP ^a^	IP, AP: OOA < YOA < YA ^a^
			(0.65)	(0.94)	(0.90)										
		R	3.76	3.30	3.00										
			(0.66)	(0.98)	(0.96)										
	AP	L	2.86	2.11	1.86										
			(0.66)	(0.58)	(0.56)										
		R	2.89	2.16	1.88										
			(0.69)	(0.62)	(0.59)										
SD of inter-tapping interval (s)	IP	L	0.03	0.04	0.04	2.27	17.72 **	8.50 **	1.33	58.23 **	116.38 **	40.12 **	IP, AP: R < L ^a^	YOA, OOA: IP < AP ^a^ L, R: IP < AP ^a^	IP: YA < YOA, OOA ^a^ AP: YA < YOA < OOA ^a^
			(0.02)	(0.03)	(0.03)										
		R	0.02	0.03	0.04										
			(0.01)	(0.02)	(0.03)										
	AP	L	0.04	0.08	0.10										
			(0.02)	(0.06)	(0.07)										
		R	0.03	0.06	0.08										
			(0.02)	(0.04)	(0.07)										

Ave: Average; Max: Maximum; SD: Standard deviation; S: Second; IP: In-phase; AP: Anti-phase; L: left; R: Right; IE: Interaction effect; ME: Main effect; YA: Young adult; YOA: Young-old adult; OOA: Old-old adult; F: F-value; ^a^: Post-hoc test of interaction effect; ^b^: Post-hoc test of main effect; * *p* < 0.05; ** *p* < 0.01.

**Table 3:** Comparison of in-phase and anti-phase tasks at phase difference.

	Task	YA (*n* = 97)	YOA (*n* = 102)	OOA (*n* = 222)	IE	ME	ME	Post-Hoc Test	
					Task × Group	Task	Group		
					F	F	F	Task	Group
SD of phase difference (degree)	IP	29.60	27.27	29.03	14.57 **	14.18 **	7.97 **	YOA, OOA: IP < AP ^a^	AP: YA < YOA < OOA ^a^
		(14.17)	(17.77)	(22.73)					
	AP	25.88	33.21	40.57					
		(12.01)	(16.13)	(22.12)					

Phase difference: Phase difference between left and right tapping; IP: In-phase; AP: Anti-phase; IE: Interaction effect; ME: Main effect; YA: Young adult; YOA: Young-old adult; OOA: Old-old adult; F: F-value; ^a^: Post-hoc test of the interaction effect; **: *p* < 0.01.

**Table 4:** Correlation analysis between bimanual coordination task and age.

	Task	Total Traveling Distance (mm)		Ave of Local Max Distance (mm)		SD of Local MaxDistance (mm)		Slope of Approximate Line of Local Max Points (mm/s)		Number of Taps(Taps)		Ave of Tapping Interval (s)		Frequency of Taps (Hz)		SD of Inter-Tapping Interval (s)		SD of Phase Difference (Degree)
		L	R	L	R	L	R	L	R	L	R	L	R	L	R	L	R	
Coefficient	IP	−0.04	0.03	0.17 **	0.23 **	−0.05	0.09	0.03	0.09	−0.34 **	−0.32 **	0.31 **	0.30 **	−0.34 **	−0.32 **	0.22 **	0.25 **	−0.01
	AP	−0.16 **	−0.08	0.30 **	0.38 **	0.12 *	0.22 **	0.03	−0.03	−0.56 **	−0.54 **	0.44 **	0.43 **	−0.55 **	−0.53 **	0.35 **	0.30 **	0.30 **

Ave: Average; Max: Maximum; SD: Standard deviation; S: Second; Phase difference: Phase difference between the left and right-hand tapping; IP: In-phase; AP: Anti-phase; L: left; R: Right; * *p* < 0.05; ** *p* < 0.01.

**Table 5:** The area under the curve, cut-off values, confidence interval, sensitivity, specificity, and Youden index for the number of taps, average of tapping interval, and frequency of taps.

	Task	Hand	AUC		*p*-Value	Cut-Off Value	95% CI		Sn	Sp	YI
			Value	SD		(Years)	Lower	Upper	(%)	(%)	
Number of taps (taps)	IP	L	0.68	0.026	<0.01	72.5	0.63	0.74	77.5	54.8	0.32
		R	0.68	0.026	<0.01	72.5	0.63	0.73	77.0	54.8	0.32
	AP	L	0.73	0.025	<0.01	65.5	0.68	0.78	93.8	44.8	0.39
		R	0.73 *	0.026	<0.01	68.5	0.68	0.78	89.0	50.5	0.40
Ave oftapping interval (s)	IP	L	0.67	0.026	<0.01	72.5	0.62	0.72	81.3	51.5	0.33
		R	0.68	0.026	<0.01	72.5	0.63	0.73	81.4	51.7	0.33
	AP	L	0.72	0.025	<0.01	73.5	0.67	0.77	78.4	57.6	0.36
		R	0.72 *	0.025	<0.01	73.5	0.67	0.77	78.7	58.0	0.37
Frequency oftaps (Hz)	IP	L	0.68	0.026	<0.01	72.5	0.63	0.73	77.3	54.3	0.32
		R	0.68	0.026	<0.01	72.5	0.63	0.73	77.2	54.8	0.32
	AP	L	0.72 *	0.026	<0.01	65.5	0.67	0.77	93.8	44.6	0.38
		R	0.71	0.026	<0.01	70.5	0.66	0.77	84.8	52.9	0.38

Ave: Average; S: Second; IP: In-phase task; AP: Anti-phase task; L: left; R: Right; AUC: Area under the curve; SD: Standard deviation; CI: Confidence interval; Sn: Sensitivity; Sp: Specificity: YI: Youden index; *: parameter with the highest AUC.

## Data Availability

The data supporting the findings of this study are available upon request from the corresponding author. The data are not publicly available because they contain information that can compromise the privacy of the research participants.
